# Intermittent exposure to high-altitude work: nocturnal hypoxemia sea-level anthropometric and biochemical markers

**DOI:** 10.1007/s11325-026-03751-7

**Published:** 2026-07-04

**Authors:** Elisa Perger, Javier Cantuarias, Sara Ottolenghi, Morin Lang, Andrea Faini, Grzegorz Bilo, Marta Pellizzari, Simona Bertoli, Carolina Lombardi, Gonzalo Araya, Perez Oscar, Gianfranco Parati

**Affiliations:** 1 Sleep Disorders Center & Department of Cardiology, Istituto Auxologico Italiano, IRCCS, San Luca Hospital, Milan, Italy; 2 Department of Medicine and Surgery, University of Milano-Bicocca, Milan, Italy; 3 Doña Ines de Collahuasi Mine Company, Iquique, Chile; 4 Department of Emergency, San Carlo Borromeo Hospital, ASST Santi Paolo e Carlo, Milan, Italy; 5 Department of Physical Therapy, Faculty of Medicine, University of Chile, Santiago, Chile; 6 Department of Electronics Information and Bioengineering, Politecnico di Milano, Milan, Italy; 7 Obesity Unit and Laboratory of Nutrition and Obesity Research, Department of Endocrine and Metabolic Diseases, IRCCS Istituto Auxologico Italiano, Milan, Italy; 8 International Center for the Assessment of Nutritional Status and the Development of Dietary Intervention Strategies (ICANS-DIS), Department of Food, Environmental and Nutritional Sciences (DeFENS), University of Milan, Milan, Italy

**Keywords:** Chronic intermittent hypobaric hypoxia, High altitude, Sleep‑disordered breathing (SDB), Central sleep apnea, Oxygen desaturation index (ODI), Obesity

## Abstract

**Introduction:**

Workers native to sea level and employed in Chilean Andes mines (3800–4500 m) experience Chronic Intermittent Hypobaric Hypoxia (CIHH) due to 7-day high-altitude shifts alternating with 7-day sea-level rest. Hypobaric hypoxia can exacerbate Sleep-Disordered Breathing (SDB), raising cardiovascular risk. Evaluating miners before altitude exposure may help identify SDB risk factors. We described nocturnal respiratory alterations during CIHH and the anthropometric and biological characteristics measured at sea level.

**Method:**

In this Cross-sectional study, nocturnal oximetry was performed in miners during high-altitude shifts. Blood samples, blood pressure, and anthropometric data were collected at sea level before ascent. Oxygen Desaturation Index (ODI) ≥ 15/h and time spent with SpO_2_ < 85% were used as markers of SDB.

**Results:**

Over 36 months, 762 workers underwent nocturnal oximetry; 30 were female, mean age 45 ± 11 years, mean BMI 27 ± 3 kg/m². 203 workers had ODI ≥ 15/h, and 559 showed nocturnal saturation < 85% at altitude. Those with higher ODI had higher BMI, total cholesterol, triglycerides, uric acid, and blood pressure at sea level. Lowest SpO₂ values were described in miners with highest BMI, uric acid, and blood pressure. BMI remained the principal independent correlate of SDB.

**Conclusion:**

Obesity may represent a marker of vulnerability to nocturnal hypoxemia in workers exposed to CIHH Preventive strategies—such as nutritional improvement, sleep apnea screening, and appropriate treatment—are essential to improve health and working conditions under CIHH.

**Supplementary Information:**

The online version contains supplementary material available at 10.1007/s11325-026-03751-7.

## Introduction

High-altitude environments represent a substantial physiological stress for individuals who are native to sea level [1]. While compensatory responses such as increased ventilation are activated shortly after ascent, acclimatization is often incomplete and highly heterogeneous [2]. In occupational settings involving recurrent exposure to high altitude, this incomplete adaptation may lead to persistent hypoxic stress, which can also manifest during sleep and negatively affect sleep quality, daytime functioning, and workplace safety [3–5].

In recent decades, Chile has experienced a marked expansion of high-altitude work activities, particularly in the mining sector [2], where individuals are continuously exposed to environmental challenges for prolonged periods. Due to their shift schedules, miners alternate repeatedly between periods of work at high altitude and rest at sea level. This pattern results in a distinct form of exposure to hypobaric hypoxia known as Chronic Intermittent Hypobaric Hypoxia (CIHH) [6]. Typically, miners follow 7-day shifts at high altitude, followed by 7 days of rest at sea level. The physiological response to hypobaric hypoxia varies among individuals, depending on genetic background, lifestyle habits, nutritional status, and anthropometric characteristics. Moreover, persistent CIHH exposure may negatively affect baseline health conditions [7, 8]. Well-documented physiological responses in miners exposed to CIHH include elevated blood pressure (BP), pulmonary hypertension, respiratory impairment, increased cardiovascular risk, polycythemia, insulin resistance, increased oxidative stress due to excessive reactive oxygen species production, and altered sleep patterns [2, 9]. Ambient hypoxia can further exacerbate sleep-disordered breathing (SDB) in susceptible individuals, worsening associated cardiorespiratory diseases [10]. Sleep apneas, both central and obstructive, induce intermittent hypoxia and sleep fragmentation, known triggers of sympathetic nervous system activation, increased BP and heart rate, with detrimental consequences for cardiovascular and metabolic systems [11]. Concerns have been raised that the combined effects of intermittent hypoxia superimposed on hypobaric hypoxia during high-altitude exposure may place individuals at an increased risk of adverse health outcomes, particularly when exposure is prolonged or chronic. Previous high-altitude studies have shown sleep fragmentation, markedly reduced mean nocturnal oxygen saturation and a substantial increase in the number of apneas or hypopneas, largely due to the emergence of central events [5, 10, 12, 13]. The cardiovascular consequences of untreated sleep apnea associated with high hypoxic burden further contribute to nocturnal arrhythmias and cardiovascular disease [14, 15].

Although the effects of high altitude on sleep and breathing have been extensively investigated in healthy volunteers and mountaineers, data from large cohorts of workers chronically exposed to CIHH remain limited. In particular, little is known about the clinical and biological characteristics associated with nocturnal hypoxemia in real-world occupational settings, where routine medical surveillance may provide an opportunity to identify individuals at greater risk.

We conducted a retrospective study at a high-altitude mining facility in Chile to characterize nocturnal respiratory patterns in individuals exposed to CIHH and to explore their association with routinely collected clinical and laboratory data obtained at sea level.

## Methods

### Study population

This study is based on a retrospective cross-sectional analysis of data collected from workers at a mine located in the Chilean Andes at high altitude (3,800–4,500 m above sea level). Most miners followed a 7-days-on/7-days-off schedule between high altitude and sea level and had been employed at the mining site for at least one year. Participants were high-tonnage truck operators commuting by company buses, working 12-hour rotating shifts at 4,100–4,500 m, and resting at a 3,800 m camp with scheduled pre-shift rest. Workers rested in individual rooms within permanent camp facilities, where overnight oximetry was performed.

In accordance with guidelines from the Chilean Ministry of Health, workers undergo periodic medical evaluations during their rest periods at sea level, including blood tests and clinical assessments. At the mine where the study was conducted, workers also undergo routine nocturnal oxygen saturation monitoring at high altitude to determine whether relocation to oxygen-enriched sleeping rooms is required.

All participants were born, raised, and permanently residing at sea level, and none were residents of moderate or high altitude (≥ 1,000 m) or belonged to high-altitude native populations. For the present study, we included only workers without known comorbidities—such as respiratory diseases or hypertension—and who were not receiving treatment for hypertension or dyslipidemia. Data were analyzed from oximetry recordings performed under room-air conditions, without supplemental oxygen, to accurately assess the nocturnal response to hypobaric hypoxia.

The study adhered to the principles of the Declaration of Helsinki and received ethical approval from the appropriate institutional authority. All participants provided informed consent for the use of their clinical data and sleep test results for research purposes.

### Study design and measures of outcomes

All miners who underwent nocturnal oximetry at high altitude (3800 m over sea level) between 2017 and 2019 were included in this retrospective analysis. Oximetry recordings were obtained during the second night of the sleep period at high altitude using a finger pulse oximeter (Nonin WristOx2 3150, Nonin Medical Inc., USA). Oximetry recordings were performed during the workers’ scheduled sleep period. These results were matched with blood test data and anthropometric measurements collected at sea level within the three months preceding the corresponding high-altitude assessment, as part of the routine occupational health surveillance schedule. A single overnight oximetry recording was analyzed for each worker.SDB was estimated based on the presence of events defined by the oxygen desaturation index (ODI) and by nocturnal desaturation, the latter identified as a mean oxygen saturation (SpO₂) < 85%. For the estimate of ODI we considered a drop of > 4% [16] according to oximeter characteristics and the presence of an ODI ≥ 15 was used as threshold to define SDB in our study [17]. As sea-level oximetries are not routinely performed before the ascent to high altitude we could not include this information in the evaluation of our cohort.

Vital signs, anthropometric characteristics—including neck circumference—and information on working habits were collected at sea level before the miners ascended for their high-altitude shifts. Blood samples were also obtained at sea level in accordance with the clinical protocols of the Mutual de Seguridad Agency. Specifically, a fasting blood sample was drawn between 08:30 and 09:00 AM for the measurement of complete blood count, blood glucose, triglycerides, total cholesterol, low-density lipoprotein (LDL) cholesterol, high-density lipoprotein (HDL) cholesterol, gamma-glutamyl transferase (GGT), and creatinine. Anthropometric measurements were obtained following international guidelines [18]. Weight was measured using an electronic scale with 100 g accuracy (Seca 700, Seca Corporation, Hamburg, Germany), and height was measured using a vertical stadiometer with 0.1 cm accuracy. Neck circumference was measured at mid-neck, between the mid-cervical spine and the mid-anterior neck, on subjects standing upright and facing forwards, with shoulders relaxed.

### Statistical analysis

Continuous variables are reported as mean and standard deviation (SD) or as median and interquartile range (IQR) for non-normally distributed data. Categorical variables are presented as absolute and relative frequencies. For the main analyses, two dichotomous variables were considered: (i) ODI < 15/h vs. ODI ≥ 15/h, and (ii) percentage of the recording period spent with SpO₂ < 85% (≥ 50% vs. < 50%). Comparisons of continuous variables between groups were performed using the t-test or the Wilcoxon rank-sum test, as appropriate. Categorical variables were compared using the Chi-square test or Fisher’s exact test, depending on expected cell counts. To investigate potential factors associated with the nocturnal respiratory response to hypobaric hypoxia, prevalence ratios and corresponding 95% confidence intervals (95% CI) were estimated using two separate log-binomial regression models—one for each outcome. In cases of convergence issues, a Poisson regression model with robust variance was applied, following Zou’s method [19]. All variables with a p-value < 0.20 in the univariate comparisons were included as covariates in the multivariable models, unless they were related to each other: (1) ODI ≥ 15/h model: covariates BMI, age, heart rate, SBP, GGT, uric acid, total cholesterol, triglycerides; (2) percentage of the recording period spent with SpO₂ < 85% (≥ 50%) model: covariates BMI, age, DBP, hemoglobin, uric acid, total cholesterol. All statistical analyses were performed using SAS version 9.4 (SAS Institute Inc., Cary, NC). A two-sided p-value < 0.05 was considered statistically significant and p-values were not adjusted for multiplicity.

## Results

**Fig. 1:**
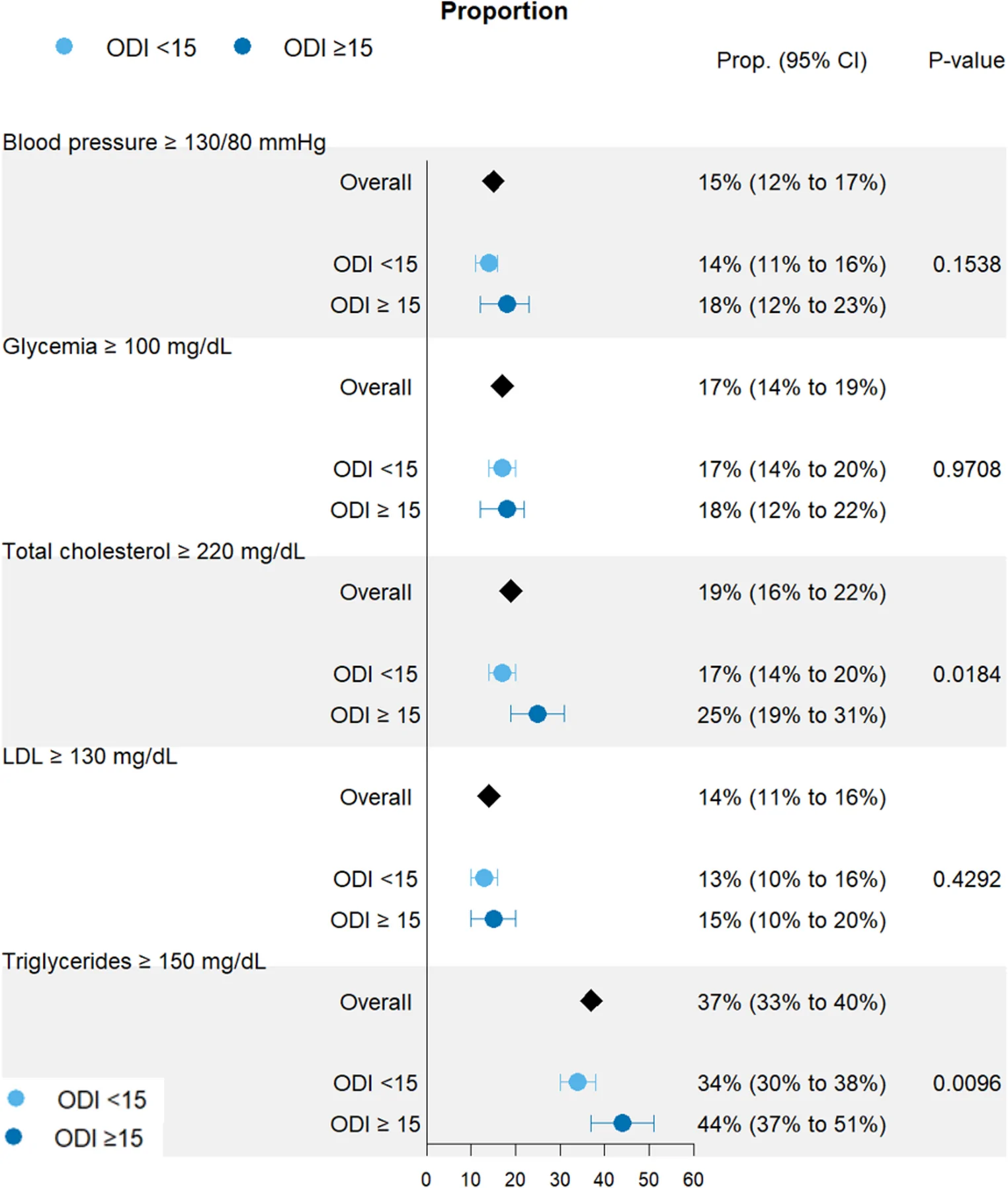
Proportion of elevated blood pressure, glycemia, total cholesterol, LDL cholesterol, and triglycerides and corresponding 95% confidence intervals (95% CI) in the overall sample (black diamond), in individuals with ODI < 15 (green dot), and in those with ODI ≥ 15 (red square)

Over a 36-month period, 1200 nocturnal oximetries were performed at high altitude. 205 were excluded due to artifacts determining less than 4 h valid exam. Finally, 762 workers presented valid nocturnal oximetry at high altitude and completed blood pressure measurements and blood sampling at sea level. Of these, 30 were female. The mean age of the population was 44.7 ± 10.7 [interquartile ranges 39.0–52.0] years, and the mean BMI was 27.1 ± 3.3 [interquartile ranges 25.1–29.1] kg/m². A total of 111 participants (15%) had systolic BP (SBP) > 130 mmHg and/or diastolic BP (DBP) > 80 mmHg, and only one worker met criteria for hypertension with SBP > 140 mmHg and/or DBP > 90 mmHg [18]. 16% (*n* = 127) had fasting glucose levels > 100 mg/dL. Regarding dyslipidemia, 19% (*n* = 144) had total cholesterol > 220 mg/dL, and 103 workers (13%) had LDL cholesterol > 140 mg/dL. Elevated triglycerides (> 150 mg/dL) were present in 268 miners (37%). These results are summarized in Fig. 1.

As shown in Table 1, workers with ODI ≥ 15/h presented lower mean SpO₂ and SpO₂ nadir. As expected, miners presenting SDB were older, with higher BMI and larger neck circumference. The neck-to-height ratio (NHR) calculated at the ratio between neck and height in cm, was also significantly higher among the groups with high ODI and with > 50% time spent with SpO₂< 85% Table (2). Workers with high ODI also presented higher SBP and DBP, although the values remained within the normal range [20]. Regarding blood samples, uric acid, gamma glutamyl transpeptidase, total cholesterol and triglycerides were higher in those presenting higher ODI. At multivariate analyses BMI (prevalence ratio [PR] 1.07 [1.03–1.11] *p* < 0.01) and SBP ([PR] 1.01 [1.00–1.02] *p* = 0.048 remained significantly associated with an ODI > 15/h (supplemetal material Tab S1). Meanwhile, BMI (PR 1.16 [1.08–1.24] *p* < 0.01) and age (PR 1.03 [1.02–1.05] *p* < 0.01) were related to the percentage of the recording period spent with SpO₂ < 85% (≥ 50%) (supplemental material tab S2). As BMI was a relevant determinant of SDB both at sea level and at high altitude, we also divided results accordingly to BMI subgroups as shown in Table 3.

**Table 1:** Comparison of clinical, anthropometric, and biochemical variables between workers with an oxygen desaturation index (ODI) ≥ 15/h and < 15/h at high altitude

	ODI < 15	ODI ≥ 15	
*N*	559	203	
Female (n/%)	28 (5%)	2 (1%)	
Age, years	46.1 [38.2–51.9]	48.2 [41.3–53.4]	**< 0.01**
BMI, kg\m^2^	26.7 [24.9–28.4]	28.1 [26.3–29.8]	**< 0.001**
Neck circumference, cm	41 [39–42]	41 [40–43]	**< 0.001**
Neck-to-height ratio	0.237 (0.018)	0.241 (0.018)	**0.019**
ODI,/h	5.6 [3–9.1]	27.8 [19.2–45.6]	**< 0.001**
SpO₂ nadir, %	82.2 [79.3–83.8]	78.0 [73.9–80.1]	**< 0.001**
Mean nocturnal SpO₂, %	86.0 [84.25–88.6]	83.7 [81.8–85.3]	**< 0.001**
Heart rate, bpm	62 [55–70]	65 [57–72]	0.09
SBP, mmHg	110 [102–120]	114 [106–124]	**< 0.001**
DBP, mmHg	66 [59–73]	67 [61–74]	**0.041**
Uric acid. mg/dl	5.5 [4.6–6.5]	5.8 [4.8–6.6]	**0.047**
Creatinine mg/dl	0.96 [0.87–1.05]	0.99 [0.87–1.10]	0.32
Glycaemia, mg/dl	92.1 [85.1–97.0]	91.2 [85.1–98.3]	0.95
Hemoglobin, g/dl	16.5 [15.9–17.3]	16.5 [16.0–17.4]	0.48
Gamma glutamyl transpeptidase, U/l	31 [21.3–47]	35 [26–53]	**0.003**
Total Cholesterol, mg/dl	168.0 [147.1–189.8]	175.1 [149.9–199.1]	**0.013**
HDL Cholesterol mg/dl	41.6 [37.2–48.9]	43.1 [37.1–49.3]	0.47
LDL Cholesterol, mg/dl	95.4 [77.1–116.2]	100.4 [77.9–120.6]	0.13
Triglycerides, mg/dl	118.3 [86.3–174.4]	140.0 [100.1–199.4]	**< 0.001**

Data are presented as median [interquartile ranges] or mean (standard deviation). A p value < 0.05 was considered as statistically significant. *BMI* body mass index, *ODI* oxygen desaturation index, *SpO₂* pulse oximetry saturation, *SBP* systolic blood pressure, *DBP* diastolic blood pressure, *HDL* high density lipoprotein, *LDL* low density lipoprotein.

**Table 2:** Comparison of clinical, anthropometric, and biochemical variables between workers with a time spent with mean nocturnal SpO₂ < 85% for < or ≥ 50%

	< 50% time with SpO₂ < 85%	≥ 50% time with SpO₂ < 85%	
*N*	702	60	
Female (n/%)	29 (4%)	1 (2%)	
Age, years	46 [38–52]	50 [45–55.5]	**< 0.001**
BMI, kg\m^2^	26.9 [25.0–28.7]	29.2 [26.3–31.5]	**< 0.001**
Neck circumference, cm	41 [39–43]	42 [40–44]	**0.004**
Neck-to-height ratio	0.237 [0.227–0.249]	0.241 [0.233–0.253]	**0.016**
ODI,/h	7.4 [3.7–14.6]	19.9 [10.8–34.9]	**< 0.001**
SpO₂ nadir, %	81.0 [78.9–84.1]	73.1 [71.5–76.5]	**< 0.001**
Mean nocturnal SpO₂, %	85.6 [84.0–87.6]	80.3 [78.3–81.2]	**< 0.001**
Heart rate, bpm	63 [56–71]	64 [55–72]	0.67
SBP, mmHg	111 [103–121]	113 [101–128]	0.32
DBP, mmHg	66 [59–73]	66 [60.5–77.0]	0.19
Uric acid, mg/dl	5.5 [4.6–6.5]	6.1 [5.0–6.9]	**0.012**
Creatinine, mg/dl	0.97 [0.87–1.05]	1.0 [0.82–1.1]	0.92
Glycaemia. mg/dl	92.2 [85.1–97.8]	90.1 [84.5–95.9]	0.50
Hemoglobin, g/dl	16.5 [15.9–17.3]	16.6 [16.2–17.5]	0.19
Gamma glutamyl transpeptidase, U/l	32.5 [22.1–48.4]	33.0 [26.2–49.1]	0.53
Total Cholesterol, mg/dl	169.2 [147.2–192.0]	178.9 [150.1–204.5]	0.09
HDL Cholesterol, mg/dl	41.1 [36.9–48.6]	44.1 [39.9–51.1]	0.06
LDL Cholesterol, mg/dl	96.5 [77.1–116.6]	104.3 [80.7–126.7]	0.15
Triglycerides, mg/dl	124.0 [89.8–179.1]	136.0 [101.1–187.2]	0.25

Data are presented as median [interquartile ranges] or mean (standard deviation). A p value < 0.05 was considered as statistically significant. *BMI* body mass index, *ODI* oxygen desaturation index, *SpO₂* pulse oximetry saturation, *SBP* systolic blood pressure, *DBP* diastolic blood pressure, *HDL* high density lipoprotein, *LDL* low density lipoprotein.

**Table 3:** Comparison of clinical, anthropometric, and biochemical variables among workers classified as normal weight (BMI ≤ 25), overweight (25 < BMI < 30), or with obesity (BMI ≥ 30)

	BMI ≤ 25	25 < BMI < 30	BMI ≥ 30	
*n*	266	377	119	
Female (n/%)	23 (8%)	5 (1%)	2 (2%)	
Age, years	43 [33–50]	48 [42–53]	48 [42–53]	**< 0.001**
Neck circumference, cm	39 [37–41]	41 [40–43]	43 [42–45]	**< 0.001**
ODI,/h	6.3 [3.3–11.5]	9.0 [4.4–17.4]	10.9 [4.2–25.4]	**< 0.001**
SpO₂ nadir, %	82 [79–84]	81 [78–83]	79 [75–81]	**< 0.001**
Mean nocturnal SpO₂, %	86.1 [84.2–87.4]	85.2 [83.7–87.4]	83.8 [81.8–86.6]	**< 0.001**
Heart rate, bpm	61 [55–69]	63 [56–71]	65 [59–74]	**0.002**
SBP, mmHg	108 [100–117]	113 [104–121]	116 [109–126]	**< 0.001**
DBP, mmHg	63 [57–70]	67 [61–73]	68 [63–77]	**< 0.001**
Uric acid, mg/dl	5.1 [4.2–6.3]	5.7 [4.9–6.6]	6.1 [5.1–7.2]	**< 0.001**
Creatinine, mg/dl	0.95 [0.86–1.04]	0.97 [0.88–1.06]	0.99 [0.86–1.08]	0.45
Glycaemia, mg/dl	91.1 [84.2–96.7]	92.0 [86.3–97.9]	93.1 [86.2–99.2]	0.12
Hemoglobin, g/dl	16.5 [15.8–17.2]	16.4 [15.8–17.2]	16.8 [16.1–17.5]	**0.01**
Gamma glutamyl transpeptidase, U/l	29.1 [20.2–39.8]	35.1 [25.1–51.3]	34.5 [26.1–52.8]	**< 0.001**
Total Cholesterol, mg/dl	164.4 [146.1–189.3]	173.0 [149.9–195.1]	169.1 [146.9–196.1]	0.09
HDL Cholesterol, mg/dl	43.2 [37.9–50.4]	41.6 [36.8–48.1]	39.1 [35.9–47.4]	**0.007**
LDL Cholesterol, mg/dl	95.5 [77.8–117.1]	97.8 [77.1–118.0]	97.1 [77.6–114.1]	0.96
Triglycerides, mg/dl	105.8 [79.1–158.3]	132.0 [94.7–185.2]	136.2 [104.8–190.9]	**< 0.001**

Data are presented as median [interquartile ranges]. A p value < 0.05 was considered as statistically significant. *BMI* body mass index, *ODI* oxygen desaturation index, *SpO₂* pulse oximetry saturation, *SBP* systolic blood pressure, *DBP* diastolic blood pressure, *HDL* high density lipoprotein, *LDL* low density lipoprotein

## Discussion

We described nocturnal hypoxemia during high-altitude exposure in workers exposed to CIHH and explored its association with BP, anthropometric measures, and laboratory parameters assessed at sea level. Miners exposed to CIHH with higher ODI presented higher BMI, cholesterol, triglycerides and uric acid together with higher BP. Additionally, higher BMI, uric acid level and BP were described in those with lower mean and nadir SpO₂. These results suggest that workers presenting SDB with recurrent nocturnal intermittent hypoxia may represent a subgroup with a less favourable cardiometabolic profile, which may be further challenged during high-altitude hypobaric hypoxia exposure.

Recent decades have seen a notable rise in body weight among individuals engaged in intermittent work at high altitudes [8]. This is primarily due to lifestyle modifications, including reduced physical activity, sedentary behavior and the adoption of unhealthy dietary habits [21]. This occupational pattern has been previously described in the context of CIHH, and is associated with higher BMI, greater neck circumference, reduced physical activity, and poorer sleep quality [22, 23]. In this setting, the relationship between SDB and excess body weight is likely bidirectional: overweight and obesity may impair ventilatory mechanics and increase susceptibility to nocturnal hypoxemia during hypobaric hypoxia, while recurrent exposure to hypoxia and sleep fragmentation may in turn contribute to adverse metabolic changes and weight gain. Moreover, overweight and obesity are recognized predisposing factors that may negatively influence the capacity to adapt to high altitude and may increase the risk of high-altitude pulmonary hypertension. It can be reasonably assumed that miners with a high BMI are at a greater risk of developing maladaptation to high altitude exposure than those with a normal BMI [8, 24, 25]. Obesity is a detrimental determinant of systemic inflammation even under normoxic conditions [26, 27]. On the other hand, obesity is also one of the most significant risk factors for SDB, with the prevalence of sleep apnea affecting approximately 40% of the moderately overweight population and up to 90% of individuals with obesity [28–30]. Among the mechanisms contributing to upper airway collapsibility—the hallmark of obstructive sleep apnea (OSA)—there are fat deposition around the pharynx and in the abdominal region [31]. In particular, abdominal obesity may increase susceptibility to OSA by imposing greater mechanical loads on the upper airway and reducing compensatory neuromuscular responses [32]. Furthermore, reduced lung volumes associated with obesity impair pharyngeal patency and disrupt the chemical control of respiration, leading to increased ventilatory instability [33]. Diminished lung volumes exacerbate apnea-related desaturations, resulting in more pronounced drops in peripheral capillary oxygen saturation, which are further compounded by the already reduced oxygen levels due to hypobaric hypoxia [34]. Even though we couldn’t evaluate the presence of SDB at sea level, it is not surprising, thus, that workers with higher BMI presented increased SDB and spent higher time with SpO₂ <85% at high altitude. Given the absence of sea-level sleep measurements, it is not possible to determine whether these alterations were pre-existing or specifically exacerbated by high-altitude exposure.

Although BMI remains a widely used indicator of adiposity and an established risk factor for SDB, growing evidence suggests that neck circumference may be a more precise predictor. Thresholds above 43 cm in men and 41 cm in women have been associated with increased OSA risk [35], likely reflecting fat accumulation in the cervical region, which directly influences upper airway patency [36]. Neck circumference has also been associated with cardiometabolic risk independently of BMI [37]. The Neck-to-Height Ratio (NHR) has been suggested as an additional marker of upper-body adiposity [38, 39]; however, in our cohort, its variability was limited and its contribution appeared modest compared with BMI and neck circumference.

A major contributing factor is the combination of sedentary behavior and excessive caloric intake, particularly evident during rest days from mining duties. During these periods, miners typically engage in minimal physical activity yet maintain a caloric surplus, fostering weight gain and dyslipidemia [40, 41]. Studies conducted in high-altitude populations have consistently reported a high prevalence of hypercholesterolemia and hypertriglyceridemia, largely attributable to obesity and unhealthy lifestyle habits rather than to hypoxia itself [42–44].

In this context, hypertension also plays an important role. We observed that workers with higher desaturation rates exhibited elevated systolic BP values. In miners exposed to CIHH, previous studies have shown increases in 24-hour systolic (SBP) and diastolic BP (DBP) at high altitude, accompanied by a reduced nocturnal SBP dip in all participants. Notably, hypertensive individuals displayed higher nocturnal DBP than normotensives despite ongoing antihypertensive therapy [23].

Across BMI subgroups, we confirmed that individuals with higher BMI showed increased BP and poorer oxygen saturation at high altitude. The contribution of SDB to hypertension is linked to intermittent hypoxia, sleep fragmentation, and intrathoracic pressure swings, which together trigger inflammation and sympathetic overactivity—mechanisms also underlying other adverse cardiovascular outcomes. Intermittent hypoxia can further promote vascular and systemic inflammation and heightened sympathetic activation [45]. In addition to its cardiovascular effects, OSA has been consistently associated with metabolic dysfunction, including insulin resistance, impaired glucose metabolism, dyslipidemia, and an increased risk of type 2 diabetes. These metabolic alterations are thought to arise from the same pathophysiological mechanisms triggered by recurrent nocturnal respiratory disturbances, ultimately contributing to a less favorable cardiometabolic profile [46, 47]. Hypobaric hypoxia itself is associated with molecular alterations, including oxidative stress, persistent systemic inflammation, generation of reactive oxygen species (ROS), and increased sympathetic activity [48]. ROS interact with LDL to form oxidized LDL, which damages endothelial cells and initiates a chemotactic cascade leading to macrophage activation and foam cell formation. The accumulation of foam cells represents the first step in the development of fatty streaks, precursors of atheromatous plaques [49]. In our population, uric acid levels were higher in participants with greater ODI and lower average oxygen saturation. Hyperuricemia contributes to inflammation, endothelial dysfunction, vascular smooth muscle cell proliferation, and renin–angiotensin system activation [50], thereby amplifying the cardiovascular effects of CIHH.

Several limitations must be acknowledged. First, the workforce was predominantly male, limiting the ability to assess potential sex-related differences in nocturnal respiratory responses to high-altitude exposure. Second,, the absence of nocturnal oximetry data at sea level represents a limitation inherent to the retrospective design of this study and to the reliance on routine occupational health assessments performed exclusively during high-altitude exposure. This important limitation precludes any causal inference and limits the interpretability of the multivariable analyses. At the same time, this retrospective design reflects real-world clinical practice, highlighting the need for nocturnal evaluation also at sea level in these workers. Third, our assessment of SDB relied on nocturnal pulse oximetry, which does not allow for precise characterization of respiratory events. A full nocturnal polygraphy would enable differentiation between obstructive and central sleep apnea, providing important diagnostic information. Identifying the specific type of sleep apnea is crucial for determining the most appropriate management strategy for workers exposed to CIHH both at sea level and at altitude, and should be addressed in future research. In addition, no objective measures of sleep duration or quality were available. Moreover, the lack of detailed information on the cumulative duration of high-altitude exposure beyond the minimum employment period may limit the assessment of potential dose–response effects of chronic intermittent hypobaric hypoxia. Finally, the absence of waist and hip circumference measurements prevented a more comprehensive assessment of central adiposity.

In conclusion, this study characterizes workers chronically exposed to CIHH, highlighting how baseline BP, anthropometric measures, and metabolic parameters are important parameter to take under consideration when dealing with hypobaric hypoxia conditions. Higher BMI was associated with impaired nocturnal oxygenation and increased oxygen desaturation events, underscoring overweight and obesity as key modifiable markers of increased vulnerability in this population.

Preventive measures—particularly those targeting obesity through improved nutrition, healthier eating behaviours, and regular physical activity—could substantially enhance the health and working conditions of miners exposed to CIHH [51]. Our findings address the need for systematic screening of SDB using nocturnal cardiorespiratory polygraphy, which would allow accurate identification and management of OSA, thereby reducing sleep fragmentation and lowering the risk of work- and driving-related accidents. Although sleep quality and lifestyle behaviours were not assessed here, future research should investigate whether these factors influence tolerance to CIHH and mitigate the risk of chronic non-communicable diseases. Additional studies are also needed to evaluate the long-term consequences of CIHH on undiagnosed sleep apnea, as well as to determine the effectiveness of sleep apnea treatments in reducing the metabolic and cardiovascular risks associated with chronic intermittent hypobaric hypoxia.

## Supplementary Information

Below is the link to the electronic supplementary material.

Supplementary File 1 (DOCX 28.7 KB)

## Data Availability

available on reasonable request.
