# Supplementary material for: Intermittent exposure to high-altitude work: nocturnal hypoxemia sea-level anthropometric and biochemical markers
Source: Sleep Breath. 2026 Jul 4;30(4):208. doi: 10.1007/s11325-026-03751-7 (PMC13332975; doi:10.1007/s11325-026-03751-7)
Supplement: Supplementary file 1 — (DOCX 28.7 KB) [file 11325_2026_3751_MOESM1_ESM.docx]

**Intermittent exposure to high-altitude work:**

**nocturnal hypoxemia and sea-level anthropometric and biochemical markers**

Elisa Perger^1,2^, Javier Cantuarias^3^, Sara Ottolenghi^1,4^, Morin Lang^5^, Andrea Faini^1,6^, Grzegorz Bilo ^1,2^, Marta Pellizzari ^7^, Simona Bertoli ^7,8^, Carolina Lombardi ^1,2^, Gonzalo Araya^3^, Perez Oscar^3^, Gianfranco Parati ^1,2^

From the ^1^Istituto Auxologico Italiano, IRCCS, Sleep Disorders Center & Department of Cardiology, San Luca Hospital, Milan, Italy; ^2^Department of Medicine and Surgery, University of Milano-Bicocca, Milan, Italy; ^3^ Doña Ines de Collahuasi Mine Company, Iquique, Chile, ^4^ Department of Emergency, San Carlo Borromeo Hospital, ASST Santi Paolo e Carlo, Milan, Italy, ^5^ Department of Physical Therapy, Faculty of Medicine, University of Chile,Santiago, Chile;  ^6^ Department of Electronics Information and Bioengineering, Politecnico di Milano  ^7^ Obesity Unit and Laboratory of Nutrition and Obesity Research, Department of Endocrine and Metabolic Diseases, IRCCS Istituto Auxologico Italiano, Milan, Italy; ^8^International Center for the Assessment of Nutritional Status and the Development of Dietary Intervention Strategies (ICANS-DIS), Department of Food, Environmental and Nutritional Sciences (DeFENS), University of Milan, Milan, Italy;

**Corresponding Author:** Elisa Perger

University of Milan-Bicocca

Sleep Disorders Center, San Luca Hospital

Istituto Auxologico Italiano IRCCS , 20149 Milan, Italy

Tel: +3902619112705

Email: [elisaperger@hotmail.com](mailto:elisaperger@hotmail.com)

[Elisa.perger@unimib.it](mailto:Elisa.perger@unimib.it)

**Supplemental material**

*Supplementals results*

Detailed multivariable analyses are provided in this Supplementary Material. Supplementary Table S1 presents the multivariable model evaluating factors independently associated with an Oxygen Desaturation Index (ODI) ≥ 15 events/h, whereas Supplementary Table S2 reports the multivariable model evaluating factors associated with spending ≥50% of the recording period with SpO₂ < 85%. Effect estimates, confidence intervals, and significance levels for all variables included in the final models are reported.

Table S1: Multivariable model of variables associated with ODI ≥ 15/h

|  | *PR (95% CI)* | *P-value* |
| --- | --- | --- |
| *Age* | *1.01 (1.00 to 1.02)* | *0.1275* |
| *BMI* | *1.07 (1.03 to 1.11)* | *0.0005* |
| *Heart rate* | *1.00 (0.99 to 1.01)* | *0.5652* |
| *Systolic Blood Pressure* | *1.01 (1.00 to 1.02)* | *0.0488* |
| *Uric Acid* | *0.98 (0.92 to 1.05)* | *0.6195* |
| *GGT* | *1.00 (1.00 to 1.00)* | *0.1781* |
| *Total Cholesterol* | *1.00 (1.00 to 1.01)* | *0.3777* |
| *Triglycerides* | *1.00 (1.00 to 1.00)* | *0.1361* |

Abbreviations: PR, prevalence ratio; CI, confidence interval; BMI, body mass index; ODI, oxygen desaturation index; GGT, gamma glutamyl transpeptidase

Table S2: Multivariable model of variables associated with spending ≥50% of the recording period with SpO₂ < 85%

|  | *PR (95% CI)* | *P-value* |
| --- | --- | --- |
| *Age* | *1.03 (1.02 to 1.05)* | *<.0001* |
| *BMI* | *1.16 (1.08 to 1.24)* | *<.0001* |
| *Diastolic Blood Pressure* | *1.00 (0.97 to 1.03)* | *0.9184* |
| *Uric Acid* | *1.04 (0.96 to 1.13)* | *0.3214* |
| *Hemoglobin* | *1.00 (0.79 to 1.26)* | *0.9919* |
| *Total Cholesterol* | *1.00 (1.00 to 1.01)* | *0.3319* |

Abbreviations: PR, prevalence ratio; CI, confidence interval; BMI, body mass index; SpO₂, peripheral oxygen saturation.
